# The Dysregulation of MicroRNAs in the Development of Cervical Pre-Cancer—An Update

**DOI:** 10.3390/ijms23137126

**Published:** 2022-06-27

**Authors:** Pui-Wah Choi, Tin Lun Liu, Chun Wai Wong, Sze Kei Liu, Yick-Liang Lum, Wai-Kit Ming

**Affiliations:** 1 Department of Research and Development, WomenX Biotech Limited, Hong Kong Science and Technology Park, Tai Po, Hong Kong; wah@womenx.net (P.-W.C.); oscarwong123@gmail.com (C.W.W.); sabrinaliu9822@gmail.com (S.K.L.); lum@womenx.net (Y.-L.L.); 2 International School, Jinan University, Guangzhou 510632, China; alanlun19981102@stu2020.jnu.edu.cn; 3 Department of Infectious Diseases and Public Health, Jockey Club College of Veterinary Medicine and Life Sciences, City University of Hong Kong, Hong Kong

**Keywords:** cervical pre-cancer, microRNA, cervical cancer, biomarkers, CIN, SIL, HPV, DNA damage response, DDR, miR

## Abstract

Globally in 2020, an estimated ~600,000 women were diagnosed with and 340,000 women died from cervical cancer. Compared to 2012, the number of cases increased by 7.5% and the number of deaths increased by 17%. MiRNAs are involved in multiple processes in the pathogenesis of cervical cancer. Dysregulation of miRNAs in the pre-stage of cervical cancer is the focus of this review. Here we summarize the dysregulated miRNAs in clinical samples from cervical pre-cancer patients and relate them to the early transformation process owing to human papillomavirus (HPV) infection in the cervical cells. When HPV infects the normal cervical cells, the DNA damage response is initiated with the involvement of HPV’s E1 and E2 proteins. Later, cell proliferation and cell death are affected by the E6 and E7 proteins. We find that the expressions of miRNAs in cervical pre-cancerous tissue revealed by different studies seldom agreed with each other. The discrepancy in sample types, samples’ HPV status, expression measurement, and methods for analysis contributed to the non-aligned results across studies. However, several miRNAs (miR-34a, miR-9, miR-21, miR-145, and miR-375) were found to be dysregulated across multiple studies. In addition, there are hints that the DNA damage response and cell growth response induced by HPV during the early transformation of the cervical cells are related to these miRNAs. Currently, no review articles analyse the relationship between the dysregulated miRNAs in cervical pre-cancerous tissue and their possible roles in the early processes involving HPV’s protein encoded by the early genes and DNA damage response during normal cell transformation. Our review provides insight on spotting miRNAs involved in the early pathogenic processes and pointing out their potential as biomarker targets of cervical pre-cancer.

## 1. Introduction

Early identification of pre-cancerous lesions and understanding risk factors are paramount in preventing cervical cancer. Statistics show that cervical cancer incidence and death rates have dropped by more than 50% in the US since the mid-1970s, partly due to increased screening which allows cervical changes to be detected before they become cancerous [1]. Although the launch of the human papillomavirus (HPV) vaccine protects women against cervical cancer, exposure to HPV before vaccination and HPV infection types that are not covered by the vaccine lead to low effectiveness of vaccination [2,3]. Many women are only protected by bivalent and quadrivalent HPV vaccines, and they may have sexual activities before taking the vaccination. The Pap test is still the major method for protecting them against cervical cancer.

The development of cervical cancer is a multifactorial, multi-step process involving the transformation of the normal cervical epithelium into cervical intraepithelial neoplasia (CIN) or squamous intraepithelial lesion (SIL), which is a non-invasive and pre-malignant lesion, which then progresses into invasive cervical cancer. SIL is used to describe the result of the cytology test, while CIN is used to report cervical biopsy results. The Bethesda system divides SIL into 2 grades: low-grade SIL (LSIL) and high-grade SIL (HSIL). HSIL is regarded as the most severe pre-cancerous stage, while LSIL is less serious. Karyomegaly, perinuclear halo, and binucleation (koilocytes) are presented in LSIL cervical cells; in contrast, intense binucleation and dyskaryosis in basal parabasal cells with little cytoplasm, hyperchromatic, and prominent nuclei are presented in HSIL cervical cells [4]. SIL would gradually develop into squamous cell carcinoma (SCC) [5]. Squamous cell carcinoma accounts for 70–80% of cervical cancer and is the most common type of cervical cancer [6]. Compared to the cytology test, which is only a screening test, biopsy results which classifies CIN into CIN1, CIN2, and CIN3 describe the actual changes in the cells. CIN1 belongs to low-grade CIN (LG-CIN), while CIN2 and CIN3 belong to high-grade CIN (HG-CIN). Early detection and effective treatment of LG-CIN and LSIL would significantly reduce the incidence of cervical cancer. Therefore, the current review will only focus on the cervical pre-cancer stage and not the later cervical cancer stage. 

MicroRNAs (miRNAs) participate in many pathophysiological mechanisms, for example, differentiation of stem cells [7], brain morphogenesis [8], muscle differentiation [9], and the development of cancer [10]. Research studies revealed that multiple miRNAs were associated with the progression from normal to cervical pre-cancerous stage. Comparing the expression of miRNA in normal and cervical pre-cancerous clinical tissue assists in finding the roles of miRNAs in the early stage of cervical transformation. Therefore, we first searched for research studies which included normal and CIN samples for analyzing the differential expressions of miRNAs. All studies are summarized in Section 2. HPV is the main factor driving the early changes in cervical transformation; the miRNAs which are potentially affected by HPV infection, especially in normal cervical cells, were explored in Section 3. Section 3 discusses the relationship between miRNAs and HPV’s etiological response in cervical cells. The DNA damage response (DDR) and HPV’s genome amplification are two major processes which transform the normal cervical cells into pre-cancer cells after the cells were infected with HPV. DDR by HPV, the earliest response upon HPV infection, affects host cell genome repairing, while HPV’s genome amplification controls the life cycle of HPV in the host cells. In Section 4, we relate the miRNAs which were found to be dysregulated across multiple studies of cervical pre-cancerous samples, to DDR and HPV’s genome amplification. These miRNAs are proposed to be involved in the early transformation process; hence, a panel consisting of miRNA targets discussed in Section 4 possesses high potential as a biomarker for the detection of cervical pre-cancer. 

## 2. MiRNAs and Cervical Pre-Cancer

### 2.1. Dysregulation of miRNAs in Cervical Pre-Cancer

Two systematic review articles conducted in 2015 and 2017 analyzed the dysregulation of miRNAs during cervical cancer progression [11,12]. In He’s study, studies published before October 2014 were included in a meta-analysis. When CIN1 was compared with normal tissues, a total of 12 miRNAs, 7 down-regulated and 5 up-regulated, were identified. As only mild cellular and nuclear abnormalities were found in CIN1 tissue, and the abnormalities are usually transient, a small number of miRNAs’ dysregulation is expected. The 12 miRNAs deregulated in CIN1 were found to be deregulated in CIN2 and CIN3, implying that these 12 miRNAs continuously contribute to the progression of CIN. In CIN2, the changes of miRNA expression were more significant. An additional 27 up-regulated and 8 down-regulated miRNAs were observed when compared with CIN1. In CIN3, miR-376c-3p (previously known as miR-368), was found to be the only additional down-regulated miRNA compared to CIN 2. In the second systematic review conducted by Pardini’s group [12], 24 studies from January 2010 and December 2017 were retrieved. Dysregulation of some miRNAs was repeatedly reported. In Pardini’s review, up-regulation of miR-21 on cervical cancer progression was found in five studies. Nine miRNAs were up-regulated across three studies and eight miRNAs were found to be up-regulated across two studies. For the under-expressed miRNAs, miR-218 was associated with cervical cancer progression across six studies, while miR-375 and miR-203 were associated in four studies and six miRNAs were associated in three studies.

As the two systematic reviews mentioned above already reviewed the related studies before 2017, Section 2 of our review mainly includes studies conducted between 2017 and December 2021, which reported differential expression of miRNAs in cervical pre-cancerous samples (LSIL, HSIL, CIN1, CIN2, and CIN3), except for studies which used SIL tissue, studies which are representative, and studies which were published before 2017 but were not included by two review articles. SIL tissue was rarely found in related studies; therefore, studies starting from 2015 were included in this review. We searched PubMed for studies published from 2014 to 2020, the subject species was limited to human, and the language of the searched articles was in English. We searched the articles using the following combinations of text and keywords as MeSH terms and textwords: “miRNA/microRNA” [miR], “cervical cancer”, “pre-cancerous lesions”, “Squamous intraepithelial lesion” [SIL], “cervical intraepithelial lesion” [CIN], “human papillomavirus” [HPV], and “DNA”. The contents of the abstracts or full-text articles listed were reviewed carefully during the literature search, and we scrutinized references from these articles to identify other related studies.

We compared the dysregulated miRNAs from studies published from 2017 onwards to those before 2017 (mentioned in two systematic review articles) and located the types of miRNAs which appeared to be dysregulated across multiple studies. We then compared those miRNAs with miRNAs which participated in regulating DDR and HPV genome amplification. The common miRNAs are highly likely to be those that have a significant effect on transforming normal cervical cells into cervical pre-cancerous cells.

Table 1 includes the research studies (more recent studies or those that were never mentioned although published before 2017) which were not mentioned in He and Pardini’s review articles. All these research studies reveal either dysregulation of miRNA in cervical pre-cancerous tissue or the relationship of the miRNAs in DDR and HPV’s genome amplification. If the miRNAs that appear in these research studies were mentioned in He and Pardini’s systematic reviews, the dysregulation of these miRNAs in cervical pre-cancer was observed in more than one study. Cross-referencing with He and Pardini’s systematic reviews could help (i) validate that the dysregulation observed is not an artifact but has been discovered across studies and (ii) spot different targets of the same miRNA. The miRNAs (miR-34a, miR-9, miR-21, miR-145, and miR-375), which are further discussed in later sections, are all repeatedly found differentially expressed in cervical pre-cancer, both in the old (articles cited in He and Pardini’s reviews) and the new (articles cited in our review) studies.

#### 2.1.1. Dysregulation of miRNAs Expression in SIL

In order to recognize the role of miRNAs in the initiation and early progression of cervical cancer, multiple research studies using HSIL and LSIL samples were performed [6,13,31,32,33,34,35]. They consistently concluded that dysregulation of miRNAs’ expression could explain the progression of SIL from normal cervical tissue. More recent studies utilizing SIL samples to identify dysregulated miRNAs are discussed as follows.

In the first study, Zeng et al. employed a narrow-down approach to identify a unique miRNA expression profile of cervical lesions and cervical cancer [35]. Despite being mentioned in Pardini’s systematic review, we include it as it is the only study which employed microarray on SIL tissue for finding dysregulated miRNA. MiR-9, miR-195, miR-497, miR-630, miR-199b, and miR-218 were found to be downregulated in the HSIL tissue while miR-21, miR-218, miR-630, miR-9, and miR-195 were downregulated in LSIL when compared to normal tissue. MiR-31 was upregulated in HSIL and LSIL, while miR-376a, miR-497, and miR-199b-5p were upregulated only in LSIL. MiR-218 was the most downregulated miRNA in cervical cancer, also, it reduced the migration and invasion ability of the HeLa cells. From other studies, LAMB3 was validated as a direct target of miR-218 in cervical and head and neck squamous cell carcinoma [36,37], while ROBO1 was proven as a direct target of miR-218 in gastric cancer and nasopharyngeal cancer [38,39]. ROBO1 promoted tumor cell invasion and metastasis while LAMB3 triggered migration and invasion. However, the miR-218—LAMB3 or miR-218—ROBO1 pathways were not proven to play significant roles in the early transformation of cervical cells. Indeed, the downregulation of miR-218 in LSIL was not significant when compared to the normal tissue. 

In another study, which also used cervical pre-cancerous clinical tissue as samples, miR-let-7e-3p, a family member of miR-let-7 [40], was found to be downregulated in the cervical pre-cancerous lesion when compared to the normal tissue. The relative expression levels of miR-let-7e-3p showed a progressive decrease in the normal cervix, HSIL, and cervical carcinoma [31].

Due to the variation in sample collection methods, another set of miRNAs was found in SIL with different expression in comparison to normal cervix. Quantitative Real Time PCR (qRT-PCR) on cervical cells collected through liquid-based cytology using cytobrush from subjects’ cervix revealed that miR-21, miR-182, and miR-200-c and miR-21, miR-146a, and miR-182 were significantly upregulated in HSIL and LSIL, respectively, when compared to the normal cells [13]. A progressive downregulation of miR-let-7b was observed when normal progresses to HSIL. Together with Chen’s study [31], two family members of miR-let-7, miR-let-7e-3p, and miR-let-7b were found to be downregulated throughout the cervical cancer progression. There are a total of 12 members of miR-let-7. MiR-let-7 is widely recognized as a tumor suppressor. It was reported that the development of poorly differentiated and aggressive cancers is due to the loss of miR-let-7 expression [41]. Boyerinas et al. indicated the downregulation of specific members of miR-let-7 in different human cancer types [42]. For instance, the reduced expression of miR-let-7a-2 and miR-let-7d were related to poor survival in head and neck carcinoma and ovarian cancer [42], while the metastasis increase in mucosal melanoma was due to the reduction in miR-let-7b and miR-let-7c expression, resulting in poor survival [43]. Moreover, miR-let-7d, miR-let-7a/f, miR-let-7g, and miR-98 were less expressed in human tumor cells with mesenchymal gene signature [44], while miR-let-7e inhibits rectal cancer metastasis [45].

In the fourth study, the relationship between HPV viral protein E6, p53, and miR-22 was studied. MiR-22 was found as one of the direct transcriptional targets of p53 in synovial cells [46]. E6 oncoprotein suppressed p53 protein in cervical cancer cells. Degradation of p53 was triggered by the binding of E6-E6AP with p53. [47]. The E6—p53—miR22 pathway was depicted in the study using clinical tissue and cell models. In the tissue part, fresh frozen cervical tissues, including normal, LSIL, HSIL, SCC, and laser-capture micro-dissected formalin-fixed, paraffin-embedded (FFPE) cervical samples, including HSIL and SCC, were employed in the study. Downregulation of miR-22 was presented in HSIL and SCC samples compared with normal samples. Concordantly, in micro-dissected FFPE samples, significantly lower expression of miR-22 was found in tumor regions in comparison to normal regions in HSIL. However, no differences were found between LSIL and normal samples [33]. 

Lastly, in Zubillaga-Guerrero’s study, 80 samples were collected by liquid-based cytology. The expression of miR-16-1 increased with the progression of the SIL. The expression of miR-16-1 in patients with HSIL was significantly higher than that in normal patients [6]. Nevertheless, when the expression level of miR16-1 in LSIL was compared with that in normal conditions a slight increase was identified, but it was not statistically significant. 

Surprisingly, across the five studies which classified the samples using the Bethesda system, no common differentially expressed miRNAs were observed. MiR-146a, miR-21, miR-182, miR-200c, miR-let-7b, miR-let-7e, miR-22, and miR-16-1 were not on the list of 13 upregulated miRNAs and 31 downregulated miRNAs that were identified by microarray in Zeng et al.’s study. Unexpectedly, in Okoye et al.’s study, upregulation of miR-21 was presented in HSIL cervical cells, but in Zeng’s study, expression of miR-21 did not show a significant change in HSIL compared to normal conditions, even though PCR-based methods were used in both studies for quantification. The types of samples may have contributed to the discrepancies observed. Although all the studies employed the Bethesda system to classify the subject’s clinical status, Zeng’s study utilized cervical tissue for both the microarray and qRT-PCR analysis, while Okoye and Zubillaga-Guerrero extracted RNA from the cervical cells collected from the cytology procedure for the subsequent PCR analysis. Whether the cervical tissue was micro-dissected or not also affects the results, as microdissection enriches the target cell population and offers a more reliable cell population for the downstream analysis [48]. Cervical tissue collected in Zeng’s and Chen’s studies did not undergo microdissection. Therefore, the bulk tissue may contain a heterogeneous population of cells. Moreover, the HPV status of each sample was not considered during the analysis in some studies [13,31]. Oncogenic proteins from HPV contribute to the dysregulation of the expression of miRNAs. Indeed, HPV integration was commonly found in the SIL tissue or cells [34]. Section 3 covers how HPV infection affects the expression of miRNAs in the cervical cells at the early stage of transformation. Other factors that contribute to the discrepancy of certain miRNA (i.e., miR-21) expression level in cervical pre-cancerous samples will be further discussed in Section 4. 

#### 2.1.2. Dysregulation of miRNAs Expression in CIN

Three studies that employed a large sample size (*n* = 727, *n* = 230, and *n* = 130) [15,16,18] covered samples representing the whole cervical cancer progression from normal to LG-CIN, HG-CIN to cervical cancer. These studies aimed at screening out miRNAs which participate throughout the whole oncogenic processes.

All three studies adopted a narrow-down approach. MiRNA microarray analysis was first employed as a screening process, followed by a validation process with qRT-PCR. However, the types of samples subjected to the microarray and qRT-PCR assays differed among three studies. In Liu’s study [16], archived paraffin-embedded tissues (CIN samples) and uterine cervix tissue biopsies (cervical cancers and normal cervical tissue) freshly frozen at −80 °C were used. In Zhao’s study [18], all samples were uterine cervix tissue biopsies obtained by colposcopy. In Kawai’s study, mucus samples collected from the cervix were used [15]. 

In Liu’s study [16], a total of 582 patients with CIN and cervical cancer and 145 patients of control group were enrolled. The validation process only included 16 miRNAs which showed the highest upregulation in cervical cancer samples in comparison to normal samples. Significant upregulation of six miRNAs (miR-20a, miR-92a, miR-141, miR-183, miR-210, and miR-944) was found in pre-malignant lesions when compared to normal cervical samples. However, during the progression from LG-CIN to HG-CIN, four miRNAs (miR-20a, miR-141, miR-210, and miR-944) were downregulated. This implies that these four miRNAs adopt different roles in the early and late stages of cervical lesion development. 

In Kawai’s study [15], a total of 86 cervical mucus samples (normal, *n* = 16; CIN1, *n* = 11; CIN3, *n* = 29; SCC, *n* = 19; and adenocarcinoma, *n* = 11) were subjected to miRNA microarray profiling. Adenocarcinoma is the second most-frequent type of cervical cancer. A positive correlation of the expression level of four miRNAs (miR-126-3p, miR-20b-5p, miR-451a, and miR-144-3p) and the severity of the diseases was demonstrated. An approximately 26- and 16-fold increase was observed for miR-451a and miR-144-3p, respectively, in CIN or CIN3 samples when compared to the normal control. The authors did not mention the expression of other miRNAs. When we further analyzed the miRNAs list in Table 1 of the study, according to the qRT-PCR results, we found that when CIN1 was compared to normal tissue, miR-155-5p and miR-205-5p were the two most upregulated miRNAs, while the most downregulated was miR-144-3p. When CIN3 was compared to normal tissue, the top four upregulated miRNAs were miR-451a, miR-144-3p, miR-155-5p, and miR-21-5p, while the most downregulated miRNAs were miR-let-7f-5p, miR-106a-5p, miR-17-5p, and miR-let-7c-5p. 

In Zhao’s study [18], all samples were HPV positive (HPV+ve). Differential expression of a total of 77 miRNAs were found in CIN3, normal, and SCC. The study mainly focused on the miRNAs targeting the E3 ubiquitin ligase Cullin 2 (CUL2), a key complex for HPV 16 E7-mediated degradation of the retinoblastoma protein (pRB). Therefore, they only mentioned the 17 miRNAs which potentially targeted the 3′UTR of CUL2. Significant downregulation of miR-154-5p, which binds to UTR of CUL2 was found in both CIN1 and CIN2/3 when compared to normal tissue. Digging up the microarray data in their supplementary material, when CIN3 was compared with the normal tissue, the top six upregulated miRNAs were miR-200b-5p, miR-200b-3p, miR-4417, miR-4738, miR-4783-3p, and miR-29c-5p, while the most downregulated was miR-617 followed by miR-433-3p. Only miR-4738-3p overlapped with the 17 miRNAs identified for 3′UTR binding to CUL2. Other important miRNAs participating in the initiation of cervical pre-cancerous lesions have been neglected. 

The approaches used by three studies in identifying miRNAs for downstream qRT-PCR validation may have missed the most differentially expressed miRNAs between normal and the CIN samples. All three studies selected the miRNAs by comparing their expression in the normal with that in the cancer samples; the CIN samples were omitted in the candidate selection process. Differential expression of miRNAs in CIN lesions were left out when compared to the normal tissue in this approach. The level of difference in miRNA expressions may have been narrowed or reversed when reaching the later cancer stage. Taking Liu’s study as an example, 16 miRNAs chosen from the top rows of the heat map diagram represent the most up-regulated miRNAs in cervical cancer tissue compared to the normal tissue, but not cervical pre-cancer to normal. Indeed, a significant difference in miRNA expression patterns was observed between the CINs and cancer tissue from their heat map, but further analysis was not observed.

Instead of comparing normal samples to the cervical cancerous samples to locate dysregulated miRNAs, the following two studies directly compared miRNAs differentially expressed in CIN with normal tissue. Furthermore, during the selection of the target miRNAs for the downstream studies, they did not include the results from the cancer samples. The direct comparison of CIN with normal biopsy tissue could reveal miRNAs that have higher correlation with the early oncogenesis process when compared to the three studies above.

In Cheung’s study [49], 48 HG-CIN, 51 SCC samples, and 9 normal cervical epithelium biopsy samples were collected. Microdissection was performed for the collection of pure neoplastic cells and normal epithelial cells. Quantitative RT-PCR was employed to assess the expression of 202 target miRNAs. CIN samples could be successfully segregated from normal epithelial cells through miRNA expression patterns. Upregulation of 10 miRNAs, including miR-518a, miR-34b, miR-34c, miR-20b, miR-338, miR-9, miR512-5p, miR-424, miR-345, and miR-10a, while downregulation of miR-193b and miR-203 in CIN cells in comparison to normal cervical epithelial cells was shown in the supervised clustering analysis. Unsupervised clustering analysis was able to separate CIN from normal tissue when this 12-miRNA signature was applied to an independent cohort of 24 patients for validation.

In Szekerczés et al.’s study, 667 miRNAs expressions were screened to identify miRNAs which distinguished healthy and diseased FFPE samples [19]. The four most significantly differentially expressed miRNAs (miR-20b, miR-24, miR-26a, and miR-100) in disease tissue compared to the paired healthy tissue and five other miRNAs (miR-29b, miR-99a, miR-147, miR-212, and miR-515-3p) which were selected based on a literature review, were subjected to the validation phase with 22 paired FFPE tissues (CIN2, CIN3, and the surrounding normal tissue). The expressions of miR-20b and miR-212 were elevated in CIN2 and CIN3 samples compared to normal tissues. MiR-515 showed a 4-fold downregulation in dysplasia tissue compared to normal tissue.

With a large sample size, a full spectrum of miRNAs screening in samples spanning across the progression from normal to the pre-cancerous stage, the above studies should serve as a foundation for further investigation of miRNAs’ network in transforming normal cells to pre-cancerous cells. However, the miRNAs revealed by these five studies were seldomly found expressed differentially between normal and neoplasia in the other small studies below, except for miR-203 [49,50].

In Lukic et al.’s study, the expression level of miR-551b was inversely correlated to the pathological cervical grade, from normal to CIN2-CIN3 [51]. In Han et al.’s study [17], significant downregulation of four miRNAs (miR-148-3p, miR-190a-5p, miR-199b-5p, and miR-665-3p) were found in the HPV+ve cancer (CIN III and cancer stage I group) in comparison to the control group (HPV+ve or HPV negative (HPV-ve)–normal and HPV-ve cancer). In Wilting’s study [50], the methylation state of miRNAs in paraffin-embedded specimens of the normal cervix, CIN1, CIN3, and SCC was compared by quantitative methylation-specific PCR (quantitative MSP). Significantly increased levels of methylation in CIN3 were found for miR-203 and miR-375 when compared to normal tissue. Methylation suppresses the expression of the target genes. It explains the observation on down-regulation of miR-203 in HG-CIN when compared to normal as revealed in Cheung’s study [49].

#### 2.1.3. MiRNAs as Non-Invasive Biomarkers for Detecting Cervical Pre-Cancer

Multiple changes of miRNA expressions were found in the cervical pre-cancer stage compared to the normal stage; therefore, miRNAs have a high potential to be biomarkers for detecting cervical pre-cancer. Early identification of pre-cancerous lesions is paramount in preventing cervical cancer. Three methods for screening cervical pre-cancer are currently available: HPV test, Pap test, and visual inspection with acetic acid (VIA) test. However, they are all invasive, meaning tools need to be inserted into the woman’s body which might cause discomfort. HPV and Pap tests are performed by holding the vaginal walls apart using a speculum and removing a sample of cells from the woman’s cervix using a brush, while the VIA test requires applying a dilution of white vinegar to the cervix, followed by the inspection of tissue abnormalities carried out by a health care provider [52]. 

Although screening is an effective way of preventing cervical cancer, the coverage rate in Asia remains low [53]. For example, in China, the proposed screening coverage was 80%. However, the actual screening coverage in 2010 was only 29% in urban areas and 16.9% in rural areas [53]. One reason that hinders Asian women from undertaking Pap tests or other screening tests may be their embarrassment due to the culturally perceived sexualization of cervical cancer screening [54]. On top of that, since all three methods for screening are invasive, it may cause concerns of pain or discomfort. Therefore, to further improve the screening coverage, a screening method that eliminates embarrassment and invasiveness is urgently needed. 

Research groups have tried using cervical tissue samples to diagnose cervical pre-cancer, for example Liu’s study [16] mentioned in the earlier section. However, an invasive procedure or a specialist is required for obtaining the cervical tissue. To screen out the cervical pre-cancer subjects, the development of a non-invasive or self-sampling procedure is of high priority. Therefore, in this section, only studies using non-invasive and self-sampling methods are included.

Several studies have investigated the potential of using differentially expressed miRNAs in blood and vaginal mucus as a diagnostic tool for cervical pre-cancer. Blood samples, including serum and plasma, were used in most studies to discover miRNAs as minimally invasive biomarkers for detecting cervical pre-cancer. Indeed, significant correlations were observed between serum and cervix miRNAs’ expression and levels and classes of Pap smear [13].

A narrow-down approach for identifying biomarkers for cervical cancer detection was conducted by Zhang et al. [20]. From screening a pool of 444 miRNA, the expression of miR-2861 in serum was determined to be significantly decreased in comparison to normal. When combined with the other three miRNAs (miR-16-2, miR-195, and miR-497), the area under the ROC curve (AUC) reached 0.734 in discriminating serum of CIN from healthy controls.

Other studies adopted a candidate approach. In Farzanehpour’s study, significant upregulation of miR-9, miR-192, and miR-205 were found in the sera from the cervical precancerous group compared to the normal group [22]. The AUC values in the pre-cancer and normal groups were 0.9, 0.98, and 0.75 for miR-9, miR-192, and miR-205, respectively. The sensitivity and specificity of using combined miRNAs to differentiate cervical pre-cancer from the normal group were not calculated. In Xin’s study, 126 patients with CIN and 60 healthy control subjects were enrolled. MiR-9, miR-10a, miR-20a, and miR-196a were upregulated significantly in sera from CIN patients compared to healthy controls [21]. The ROC analysis showed that this four-miRNA signature showed high accuracy (AUC = 0.886) in discriminating CIN individuals from healthy controls. In the fourth study, sera from 329 women (159 healthy women, 46 cervicitis, 46 atypical squamous cells of undetermined significance [ASCUS], 40 LSIL, 28 HSIL, and 10 SCC) were obtained [13]. Higher expressions of miR-21, miR-146a, miR-155, miR-182, and miR-200c and lower expression of miR-let-7b were observed in SIL sera than in sera from normal subjects. Furthermore, in Liu’s study, 105 patients with cervical cancer, 86 patients with CIN, and 50 healthy control subjects were enrolled. The expression level of miR-196a was higher in the serum of patients with cervical cancer and CIN than those in healthy controls. Serum miR-196a was also found to be associated with the CIN grade [55]. 

Plasma is another source of blood samples that can be easily obtained. One study utilized plasma to differentiate CIN1 with CIN2 by the expressions of miR-21, miR-214, miR-34a, and miR-200a [23]. MiR-21 progressively increased from normal to CIN1, CIN2 to CIN3, while miR-214, miR-200a, and miR-34a progressively decreased from normal to CIN1, CIN2, and CIN3. Significance in the difference in expression was only observed between normal and CIN2 or CIN3 but not with CIN1. The study further classified CIN1 and lower samples as CIN1- (including CIN1 and normal) and CIN2 or higher as CIN2+ (CIN2 and CIN3) for assessing the diagnostic power of these miRNAs. The AUC values for miR-21, miR-34a, miR-200a, and miR-214 were 0.613, 0.508, 0.615, and 0.505, respectively, for discriminating CIN- and CIN2+.

Non-blood sampling includes vaginal lavage and vaginal mucus. Lavage was collected with a self-sampling device. Bi-marker MAL (T-lymphocyte maturation-associated protein)/miR-124-2 methylation test with HPV 16/18 genotyping on the lavage samples could differentiate CIN3+ and CIN2+ from the normal group with similar sensitivity but a lower specificity than the cytology test. The sensitivity of the bi-marker test with HPV 16/18 genotyping ranges from 77.6% to 88.4%, while the specificity ranges from 29.8% to 54.8% for detecting CIN3+. For detecting CIN2+, the sensitivity ranges from 67.1% to 85.8%, and specificity ranges from 30.9% to 55%. In contrast, cytology yields a sensitivity of 77.9% and specificity of 77.6% for CIN3+ and a sensitivity of 74.3% and specificity of 81.3% for CIN2+ [56]. Although the methylation assay has lower specificity than the cytology test, it could re-attract subjects who are reluctant to undergo the cytology test but tested positive with the lavage test to undergo the cytology test after all for higher protection. 

The other study collected vaginal mucus through a cotton swab. Circulating cells and local cells, substances secreted by cervical tissues, vaginal discharge, menstrual blood, and cervical exfoliated cells were present in the cervical mucus. MiRNAs found in the cervical mucus might be secreted by any of these sources. The miRNA biomarkers discovered can be used to develop non-invasive detection methods for cervical pre-cancer when a collection device that could be placed close to the vulva for the collection of the mucus is available. Employing the vaginal mucus samples, the AUC for detecting CIN3+ was 0.80, 0.82, 0.87, and 0.87 for miR-126–3p, miR-20b-5p, miR-451a, and miR-144-3p, respectively [15]. 

The dysregulated miRNAs in cervical pre-cancer when compared to normal in clinical samples are summarized in Table 2. Combining the articles in He and Pardini’s systematic review, the source of the studies which revealed the differential miRNAs expression in cervical pre-cancer is completed. 

## 3. HPV Induced miRNA Changes in Cervical Pre-Cancer

Cervical cancer is usually correlated with oncogenic HPV positivity. The expression of miRNAs in cervical cancer could be affected by HPV viral proteins [57]. In particular, HPV infection is a crucial prediction in the risk assessment of different pre-malignant lesions [34]. The association between the dysregulation of miRNAs and HPV infection was reviewed in 2015 [58]. However, the review did not focus on the association of those in the cervical pre-cancer stage. Indeed, several studies have demonstrated that the dysregulation of miRNAs’ expression was correlated with HPV infection in cervical pre-cancer [5,6,7,8,9,12,16,19,26,28,38,39].

### 3.1. MiRNAs Expression in Cervical Pre-Cancerous Tissue Infected with HPV

This section focuses on the changes to miRNAs’ expression from normal to HPV-positive cervical pre-cancer. 

First of all, infection with high-risk HPV (hrHPV) causes dysregulation of miRNAs in normal cervical cells. 

In Liu’s study, hrHPV infection was found in 82% of HG-CIN, 64% of LG-CIN, and 24% of normal samples. MiR-20a, miR-141, miR-210, and miR-944 were significantly upregulated in the normal samples with hrHPV infection when compared to those without [16]. In another study, which aims at resolving whether miR-34a expression was induced by hrHPV, 32 normal cervical epithelia with hrHPV infection and 32 normal epithelia without hrHPV infection were gathered for analysis. Significantly lower expression of pri-miR-34a was observed in normal cervical epithelium with hrHPV infection than in those without [59]. Moreover, a study which included samples from 43 women with HPV infection but free of cancer, 46 women free of both HPV and cancer, and 9 CIN1/6 CIN2 showed that miR-21 was upregulated in HPV+ve samples with no evidence of carcinogenic changes in comparison with controls, but the findings were not significant. Downregulation of miR-29 was observed in HPV+ve samples in comparison to healthy controls, but the findings were also not significant.

Second, infection with hrHPV led to miRNAs’ dysregulation in cervical pre-cancerous tissue. 

In a small-scale study [19], HPV+ve CIN2–CIN3 samples showed 2.9-fold miR-20b upregulation and 1.15-fold miR-515 downregulation compared to the normal tissue. When eliminating the HPV state, 2.4-fold increased expression of miR-20b and 4-fold decreased expression of miR-515 were observed in CIN2–CIN3 samples in comparison to normal tissues with statistical significance. This demonstrates that HPV exerts a certain control on the expressions of miRNAs in the CIN stage. Another study showed that the downregulation of miR-34a in CIN2–3 compared to CIN1 was correlated with HPV 16 positivity [60]. The expression of miR-34a precursor (pri-miR-34a) was also found to be reduced in the CIN samples with hrHPV infection when compared to those without [59]. The relationship between E6 and pri-miR-34 will be further discussed in Section 3.3. 

Third, different HPV strains lead to different miRNA changes in the pre-cancer stage of cervical cancer. 

HPV 16 is the most frequent type of HPV among 14 cervical cancer-causing hrHPV types. In Liu’s study, among the hrHPV-positive samples, 23% of normal controls, 15% of LG-CIN, 41% of HG-CINs, and 76% of cervical cancers were HPV 16 positive. The expressions of miR-29a, miR-183, and miR-944 were higher in HG-CIN patients infected with HPV 16 than in patients with other hrHPV types. Due to the small number of HPV-negative CIN and cancer samples, the expressions of miRNA were not compared between cancer or CIN samples with and without hrHPV infection [16]. In a study that included mucus samples [15], the correlation of the level of HPV infection with the expressions of miR-451a, miR-144-3p, miR-126-3p, and miR-20b-5p was analyzed. Samples with HPV 16/18 positivity showed the highest levels of these miRNAs when compared to the HPV-negative, HPV-positive (infected with ≥1 type of HPV), 7 HPVs (HPV 16, 18, 31, 33, 45, 52, 58), and 13 HPVs (16, 18, 31, 33, 35, 39, 45, 51, 52, 56, 58, 59, and 68) groups. Next, multiple HPV infections showed different patterns of miRNAs dysregulation from single HPV infection. In CIN2–CIN3 tissue with multiple HPV infections, expressions of miR-27a and miR-34a were lower than those with a single HPV infection [60]. Once again, this demonstrates that the alterations in miRNAs’ expression is HPV strain dependent.

In addition, the expression of miRNAs in the cervical tissue might be affected by the HPV integration state. 

In a cytological screening, the SIL samples with a less-integrated HPV state were compared to the SCC samples. The SIL and SCC samples with integrated states showed a higher miR-16 expression than those with a mixed (integrated or non-integrated) state. On the other hand, SIL and SCC samples with a mixed HPV state had a ~5.5-fold higher miR-16-1 expression than the normal samples without HPV. With HPV integrated, miR-16-1 expression increases around ~7 fold when compared to HPV-negative samples. However, as all HPV-negative samples were normal samples, and after adjustment, variability in the expression level of miR-16-1 induced by SIL and SCC outweighs those by HPV physical state [6]. 

Last but not least, HPV may drive the methylation of miRNAs. The methylation of miRNAs led to a decrease in miRNAs’ expression. HPV-immortalized keratinocytes (16 E6/E7 HFK) are the reminiscence of HG-CIN. The 16 E6/E7 HFK showed an increase in methylation of miR-149 when compared to human foreskin keratinocytes [50]. However, for miR-203 and miR-638, the methylation status was not conclusive. Two out of five 16 E6/E7 HFK and two out of three 16 E6/E7 HFK showed methylation of those two miRNAs. Surprisingly, in FFPE cervical tissue, methylation levels of miR-203 and miR-375 were significantly increased in both CIN3 and SCCs. In the cervical scrape, similar results were observed. Methylation of miR-203 was elevated in the hrHPV-positive cervical scrapes of women with abnormal cytology and HG-CIN when compared with cervical scrapes of women with normal cytology [50].

The miRNAs dysregulated in clinical samples owing to HPV infections are listed in Table 3.

### 3.2. Regulation of HPV’s Viral Proteins by miRNAs

#### 3.2.1. HPV Viral Protein

The HPV genome contains three central regions: the early region (E)-encoded gene (E1, E2, E4, E5, E6, and E7), the late region (L)-encoded gene (L1, L2) for the production of the capsid protein, and the upstream regulatory region (URR). E1 and E2 initiate the replication and transcription of viral DNA. E4 helps to prolong the replication and transcription. E5 interacts with EGF receptors to promote cell growth. Finally, E6 and E7 interact with p53 and pRB to introduce carcinogenesis [61]. As some miRNAs can directly or indirectly be targeted to inhibit the transcription of HPV viral proteins, these miRNAs might be a therapy for cervical cancer. Table 4 summarizes the miRNAs which target the HPV encoded protein. 

#### 3.2.2. Regulation of HPV E1/E2 by miRNAs

The E1-E2-mediated viral replication is essential for the amplification of the HPV genome. Inhibiting E1/E2 by miRNAs could be a novel anti-HPV therapy. MiR-221 is highly expressed in viral infections, including the hepatitis C virus, human immunodeficiency virus type 1, and HPV. A recent study reported that upregulation of miR-221 was observed in HPV-infected patients’ serum and cervical cancer cell line [28]. Overexpression of miR-221 inhibited E1–E2 mediated replication. In the SiHa cell, it reduced HPV’s replication by activating the SOCS1/Type I Interferon (IFN) signalling pathway [28]. Upon infection with HPV 16, an immune response is triggered. The upregulation of miR-221 positively regulates IFN-I production and interferon-stimulated genes’ (ISGs) expression. As the author did not mention the status of the patients with HPV infection, our estimate is that the patients were at the very early cervical pre-cancer stage and an immune response was triggered to fight against the HPV infection. However, further studies which use CIN samples with HPV 16 infections for revealing the expression of miR-221, IFN, and other downstream effectors participating in HPV genome replication are required to validate this hypothesis. In addition to miR-221, miR-139-3p also targets the E1 region of HPV 16. The HPV 16 viral load and the expression of miR-139-3p were negatively correlated in head and neck cancer tissue, which suggested that miR-139-3p has a potential role in regulating HPV 16 replication during the initiation of infection with HPV 16 [62]. Apart from miR-221 and miR-139-3p, miR-145 also inhibited E1–E2 mediated replication. The seed sequence of miR-145 was present in the coding region of HPV’s E1 and E2. The overexpression of miR-145 reduced transcription factor KLF-4 in CIN-612 cells and HPV E1 protein in HPV-31 organotypic rafts. KLF-4 is a transcription factor which induces stem cell pluripotency. The downregulation of KLF-4 leads to differentiation, and both activities are important to allow for viral genome amplification upon differentiation [63]. More details about miR-145 will be mentioned in Section 4.1.2.

#### 3.2.3. Regulation of HPV E6 by miRNAs

MiR-375 was overexpressed in HPV-positive CaSki and SiHa after 5azadC treatment and could promote E6 mRNA instability. It is suggested that miR-375 is a potential inhibitor for E6 despite the fact that the molecular mechanism remains vague [26]. Overexpression of miR-375 was also found to positively regulate dicer through the downregulation of viral E6 in the cell line model. Dicer is an important miRNA processing protein that was found to be downregulated in cervical cancer [25,71]. The E6 protein was thought to induce degradation of TAp63, an isoform of the transcription factor p63 within the p53 family. It had been suggested that TAp63 is an activator of the dicer promotor, which allows dicer transcription. Therefore, the expression of miR-375 has an indirect effect on the dicer level [25]. Another study demonstrated that miR-122 and miR-409-3p also inhibit E6 [65,66]. MiR-122 bound directly to E6 and E7 mRNA and greatly inhibited E6 transcription in SiHa. For miR-409-3p, the binding site was predicted by the STarMir tool [66]. In addition, MiR-875 and miR-3144 inhibited E6 transcription by switching the E6/E6* mRNA ratio. E6* is from the spliced E6 mRNA and is not oncogenic. These miRNAs inhibited the epidermal growth factor receptor (EGFR) and promot E6 splicing [67]. Last but not least, a group from Université de Strasbourg successfully produced artificial microRNA (Ad16_1 and Ad18_2) against E6 protein in HPV 16 and 18 cancer cells. Ad16_1 targeted E6 in HPV 16 while Ad-18_2 targeted E6 in HPV 18. These miRNAs successfully knocked down the expression of E6 and induced apoptosis of HeLa and SiHa cells [64].

#### 3.2.4. Regulation of HPV E6–E7 by miRNAs

MiR-139-3p was downregulated in HPV+ve tissue. Besides targeting HPV E1 during the early infection, it was proposed that, after integration, it inhibits the production of E6 and E7 protein [62]. Other miRNAs, miR-129-5p, miR-331-3p, and miR-744, also inhibited E6 and E7 transcription by targeting proteins in host cells [51,52,53]. MiR-129-5p targeted the 3′-UTR in SP1, a transcription factor and could bind to the upstream regulatory regions of the HPV-18 genome. HeLa cells is HPV-18 positive, concordantly, overexpression of miR-129-5p inhibited E6 and E7 transcription in HeLa cells [68,69,70]. MiR-331-3p directly targeted a protein, neuropilin-2, for the inhibition of E6 and E7 transcription in cervical cancer cells. This suppression caused the arrest of cell proliferation [69]. On the other hand, miR-744 was up-regulated in SiHa cells when they were treated with lactic acid. Lactic acid is a product of glycolytic tumour cells and is associated with restricted patient survival in cervical cancer. MiR-744 binds to the promoter site of the pro-tumorigenic factor (Rho GTPase-activating protein 5) ARHGAP5, resulting in an increase ofARHGAP5. MiR-744 and ARHGAP5 together reduce the E6/E7 protein level in lactic acid-treated SiHa cells [70].

### 3.3. MiRNAs Affected by HPV Viral Proteins

Transcription factors c-Myc, p53, and E2F modulate the expressions of many miRNAs. Oncogenic HPV E6 and E7 modulate the expression of miRNAs through the transcription factors above.

Oncoproteins E6 and E7 are the most-identified proteins causing changes in miRNAs’ expression in cervical cancer. As mentioned in Section 2, miR-22 was downregulated in HSIL compared to normal samples that were without HPV infection. MiR-22 was identified as a direct transcriptional target of p53 [46]. In C33A and Hela cells, miR-22 was down-regulated by HPV 16 E6 via the E6/P53/miR-22 pathway [33]. Another miRNA controlled by p53 is miR-34a. The expression of tumor-suppressive miR-34a is inhibited by HPV 16 and HPV 18 infection through E6 destabilization of p53 [24]. By knocking down HPV 18 E6 with the E6-specific siRNA, and the ectopic expression of miR-34a in HeLa cells, an E6—p53—miR-34a—p18lnk4c axis that inhibits the CDK4 and CDK6 function was confirmed [72]. P18lnk4c is a cell cycle-regulator and tumor-suppressor protein. However, its tumor-suppressive role was not found in cervical cancer. In reverse, increased expression of p18Ink4c was observed in HPV 16+ve CIN2 lesions in comparison to normal tissue. One of the explanations for this could be the lack of a G1 checkpoint owing to the degradation of pRB caused by viral E7. Nevertheless, by employing C33A cells which lack HPV infection and express a mutant p53, a direct link between p53 and miR-34a expression was demonstrated. Ordinary C33A did not have miR-34a expression. The production of miR-34a was induced by ectopic expression of wild-type p53 in C33A [24]. 

In addition to the expression of miRNAs, hrHPV E6/E7 also affects the expression of the miRNA processing protein. When comparing HPV+ve and −ve cervical carcinoma cell lines, higher levels of DROSHA and DICER mRNA were observed in HPV+ve cancer lines. The mRNA and protein expression of DROSHA and DICER increased when ectopic hrHPV E6/E7 were expressed in the normal human epithelial cells and C33A cervical carcinoma cells which were HPV negative. Most importantly, many DROSHA-regulated miRNAs were dysregulated in HPV 16 E6/E7-expressing cells [73]. Indeed, copy number gain in chromosome 5p, where DROSHA is located, was a frequent observation in cervical cancer [74]. Murty’s group found an increase in the chromosome 5p copy number in invasive cervical cancer, which results in the overexpression of a number of its target genes, including DROSHA [74]. DROSHA significantly influenced the miRNA profiles in cervical cancer promotion and progression [75,76]. However, the precise stage of cervical cancer progression where DROSHA expression increased remains unclear. Scotto et al.’s study showed using a FISH assay that the earliest stage in disease development in which chromosome 5p gain occur is HSIL [74]. However, the alteration in DROSHA level seemed to occur after HSIL. A study by Coleman’s group showed that the DROSHA copy number increased in several cervical SCC samples but not in any SILs, neither in LSIL nor HSIL [75]. A solid conclusion about whether DROSHA overexpression occurred during malignancy or at an earlier stage in CC development was yet to be determined. Nevertheless, in Harden’s study [73], C33A cells transfected with HPV 16 or HPV 18 or E6 and E7 protein showed an increase in the expression of DROSHA mRNA. This has shed light on the possibility that the DROSHA level may elevate at an earlier stage than first envisioned.

### 3.4. MiRNAs Related to DNA Damage Response in HPV and Cervical Cancer 

Genomic instability is one of the common characteristics of cancer. It could be found at both the DNA base level and chromosomal level. It is an early event during HPV infection that antecedes the integration of viral genome into the host genome and the appearance of the pre-cancerous lesions. Failures in cell cycle checkpoints, including DNA damage checkpoints, mitotic checkpoints, and DNA repair systems, cause genomic instability. Ataxia–telangiectasia-mutated (ATM), ataxia- and Rad3-related (ATR), and DNA-dependent protein kinase catalytic subunit (DNA-PKcs) protein are involved in the response to damage at the DNA base level. ATM and DNA-PKcs are responsible for DNA double-strand break (DSB), while ATR is responsible for single-stranded DNA break (SSB). Loss of function in these protein interrupts DNA damage recognition and repair. Accumulation of the mutation drives the initiation of tumorigenesis [77]. HrHPV could activate DDR-related proteins through its viral replication proteins (E1 and E2) [78]. When hrHPV enters the host cell, E1 and E2 mediate the replication for quick generation of viral genome. The DNA generated in the quick replication forms an “onion skin” structure which causes the formation of DNA damage repair [78,79,80]. Although the exact mechanism of HPV-activated DDR is still not well understood, it is tempting to investigate the miRNAs’ regulation in the HPV-activated DDR. Therefore, miRNAs that regulate DDR-related proteins in the cervical cancer cell line were included in Section 3.4.1, Section 3.4.2 and Section 3.4.3. The studies below employed ionizing radiation or chemotherapeutic drugs, for example doxorubicin and etoposide, to trigger DNA damage. The artificial agents could induce the mismatches of DNA bases, SSB, DSBs, chemical modifications of the bases, and cross-linking of inter-strand or intra-strand [81]. Multiple miRNA changes were observed upon radiation or treatment with chemotherapeutic drugs in the cervical cancer cells. The aims of most studies were revealing the roles of miRNAs in altering the sensitivity or resistance towards radio- or chem-therapy, but not studying the DDR triggered by HPV. Although the intensity of DDR induced by the artificial agents may not be the same as the DDR introduced by HPV, it provides a good means to study the participation of miRNAs in the DDR-related pathways. These studies shed light on the relationship between HPV infection and DDR at the early stage of cervical transformation. 

#### 3.4.1. Regulation in Proteins Responsible for DDR Initiation 

ATM is the first responder of the DSB. The activation of ATM phosphorylates CHK2 (checkpoint kinase), which in turn triggers the phosphorylation of downstream molecules. These series of kinase cascades arrest the cell cycle and trigger the DNA repairing process [82]. MiR-421 suppressed the ATM mRNA by directly binding to its 3′ UTR in the HeLa cell line. This resulted in the reduction of cell arrest in the S phase [83]. On the other hand, miR-18 increased the sensitivity to radiation treatment in the SiHa and HeLa cell lines by hampering the DDR as miR-18 directly targeted the 3′UTR of ATM [84]. Furthermore, MiR-148a suppressed the expression of CDK1, a downstream effector of ATM, by directly binding to CDK1. In cervical cancer cells, the miR-148a level was reduced due to the high expression of SNHG12, the inhibitor of miR-148a [29]. The expression of CDK1 and cell growth were elevated as a result. In contrast, when miR-148a was over-expressed, it could lead to cell cycle arrest in cervical cancer.

#### 3.4.2. Regulation in DSB Marker Proteins

Phosphorylation of histone variant H2A.X on serine 139 (γH2A.X) is the earliest marker of the DNA damage. ATM phosphorylates the H2A.X’s serine residue after the DSB is recognized by the MRE11—RAD50—NBS1 (MRN) complex. Subsequently, γH2A.X activates the p53/p21 pathway and recruits DNA-repairing proteins such as MDC1 and 53BP1 to the DSB [85,86]. Therefore, γH2A.X is essential for the DNA damage response. MiR-138 showed a significant role in downregulating H2A.X. It directly targeted the 3′ UTR of H2A.X mRNA to suppress the expression of H2A.X, resulting in enhanced genomic instability and cellular sensitivity to DNA-damaging agents [87]. Its expression suppressed the H2A.X expression in the HeLa and SiHa cell lines [87]. Such an interaction may be affected by the presence of the HPV E6/E7 protein. HPV E6/E7 may reduce the interaction between miR-138 and γH2AX. In lung cancer cells treated with radiation, the HPV E6/E7 transformed lung cells’ CLR2471 showed a lesser increase in their expression of miR-138 when compared to A549 lung cancer cells without E6/E7 [88]. Therefore, the presence of HPV infection in cervical cells may hamper the interaction between miR-138 and γH2A.X. However, whether the interruption happened during the pre-cancer stage or the cancer stage was not known. In contrast, miR-34a and miR-449a induced γH2A.X by suppressing PACS-1 in HeLa. Similar to miR-138, miR-449a might be under the control of the HPV viral protein, as there was no expression in miR-449a in HPV-negative cell lines HT3 and C33A [30].

#### 3.4.3. Regulation in DNA Damage Repairing Proteins

RAD51 is a recombinase that is important for stabilizing the DSB ends. It works with RAD50, 52, and 54 to facilitate the recognition of the initiation of homologous recombination [89]. It also works as a replication fork protection and reversal enzyme to stabilize the single-stranded DNA during DNA repair [90]. REV1 is a translation DNA synthesis (TLS) polymerase that protects the genome from a large scale of deletion [91]. MiR-96 downregulated the RAD51 and REV1 gene by directly binding to their 3′ UTR. It is abundant in the HeLa cell line, and its overexpression increases the cells’ sensitivity to cisplatin [92]. MiR-4429 was associated with radioresistance in cervical cancer cells, as it was downregulated in the radio-resistant cervical cancer cells. Overexpression of miR-4429 promoted sensitivity to irradiation in cervical cancer by suppressing RAD51 [93]. 

Direct interaction between miRNAs and the protein or genes involved in HPV-related DDR in cervical pre-cancer cells or HPV-infected early-transformed cells were not found. Nevertheless, the same panel of miRNAs observed from the above studies may also participate in the DDR response related to HPV infection in the early cervical transformation stage. A cell line model for mimicking the early transformed cervical cells would help elucidate the participation of these miRNAs in the DDR response that happened in the early transformation stage. The expression of these miRNAs and the DDR proteins should be evaluated in the cervical pre-cancerous and normal tissue samples for further validation. Nevertheless, Section 4 will try to relate the dysregulation of miRNAs in cervical pre-cancer obtained in past research with HPV-related DDR. Table 5 summarizes the miRNAs associated with the DDR of cervical cells.

## 4. The miRNAs with Critical Roles in Early Transformation of Cervical Cells

We compared the miRNAs appearing in Section 2 and Section 3 with the miRNAs in He and Pardini’s reviews (Table 1). Several miRNAs were repeatedly found to be dysregulated in the cervical pre-cancerous tissue. The dysregulation observed was not due to artifact but to a true change of expression. There is a high chance that these miRNAs participated in the DDR and HPV viral transcription process that happened during the early transformation, because the relationships were already demonstrated in other types of cancer or similar cellular responses (mentioned in Section 3). These miRNAs are miR-375, miR-145, miR-34a, miR-9, and miR-21. Section 4 will discuss the roles of these five miRNAs in the transformation of normal cervical cells. 

### 4.1. miR-375, miR-145, miR-34a, miR-9, and miR-21 in HPV-Induced Cervical Pre-Cancer

#### 4.1.1. miR-375

In 2011, miR-375 was found to be downregulated in cervical pre-cancer (CIN2/3) tissue significantly for the first time [94]. In humans, the *miR-375* gene is found on chromosome 2q35 [95]. In an analysis of focal aberrations in HG-CIN samples, focal loss at chromosome region 2q35 was the most frequent loss region [96]. All samples used in the focal loss study were high hrHPV+ve with strong diffused staining for CDKN2A, indicating a transformation of HPV infection. This implies that focal loss of the 2q35 region is highly related to infection with hrHPV. However, whether it is a consequence of hrHPV infection or the loss of it driving the cells to be more susceptible to hrHPV infections was not determined. In addition to the focal loss of chromosome, another mechanism, methylation, which results in the downregulation of target miRNA (i.e., miR-375), was also found in the HPV-positive samples. Methylation of miR-375 increased in both HPV-immortalised keratinocytes when compared to the non-immortalised counterpart and CIN FFPE samples when compared to the normal samples [50]. Furthermore, the percentage of samples with miR-375 methylation increases with the stage of CIN, with the later stages showing a higher number of samples with hrHPV infections. It is highly possible that the methylation of miR-375 is positively correlated to the infection with hrHPV. Rationally, with the focal loss and methylation, downregulation of miR-375 expression should be presented in HPV-positive CIN samples. Indeed, downregulation of miR-375 was repeatedly found in the HPV-positive CIN samples, no matter whether they were in FFPE [96], frozen [94,96], or exfoliated [14,94] samples. From normal cervical epithelium to CIN to SCC, the expression of miR-375 decreased step by step [14,96]. In 2021, a study that recruited 9972 women again confirmed miR-375 downregulation in CIN2/3 samples in comparison with normal tissue [97]. Due to the high relatedness between miR-375 and hrHPV infection, the detection of miR-375 expression level in HPV+ve exfoliated samples achieved a higher AUC than the Pap test in identifying CIN2 and CIN3 with HPV+ve [14]. Detection of miR-375 level was more sensitive than cytology in determining CIN2/3 in HPV 16-positive samples [97]. However, when the level of miR-375 was used for the identification of CIN2/3 in samples with other hrHPV infections (besides HPV 16/18), the result was just comparable to cytology. This implies that HPV 16 infection is more related to the dysregulation of miR-375 in cervical cells when compared to infections with other hrHPV types. As mentioned in Section 3.1, miR-375 downregulated the expression level of E6. The loss of miR-375 would increase the expression of E6, leading to more inactivation of p53 tumour-suppressor genes. MiR-375 indeed functions as a tumour-suppressor gene [98]. p53 is inactivated by the loss of miR-375, leading to uncontrolled cell proliferation and resistance to cell death [99]. In addition, E6 increases mTORC1-dependent cellular growth and proliferation [100] and activates the hTERT promoter to establish replicative immortality [101]. Taken together, this leads to uncontrolled growth of the cervical cells. Aligning with the above axis, in an in vitro study, miR-375 was found to suppress cell viability in SiHa and CaSki cells [96]. In addition to the pathway above, miR-375 was recently found to affect cervical cells’ proliferation and causes cell cycle arrest through the miR-375—AEG-1 axis [102] and miR-375—IGF-1R axis [103]. It was repeatedly proven that the loss of miR-375 was found in cervical cancer cell lines and different types of clinical tissue. Considering all the evidence above, the potential of miRNA 375 to initiate the early transformation of cervical cells by acting on E6 protein is worth studying.

#### 4.1.2. miR-145

In Section 3.2.2, we mentioned that miR-145 targeted transcription factor KLF-4 in CIN-612 cells and HPV E1 protein in HPV-31 organotypic rafts resulting in the suppression of E1–E2-mediated replication. E1 is a helicase responsible for unwinding the viral replication origin to recruit host cellular factors for the subsequent viral genome replication. [104]. In cells which have E1 protein overexpressed, ATM and ATR pathways, which are involved in the DDR, are induced. Upregulation of phosphorylated Chk2 and γH2AX indicates the activation of ATM while phosphorylated Chk1 indicated that the ATR pathway was induced [104]. As a result, the cell cycle is arrested, and the DNA repairing process is started. At the same time, HPV utilizes the component in the ATM pathway for viral genome amplification [105]. The expression of miR-145 in cervical pre-cancerous cancer samples were revealed in six studies. However, two studies (by Shi 2013 and Guo) which were mentioned in He’s systematic review were not searchable [11]. In the other four studies [37,94,106,107], all four included microarray data and two included qPCR data on normal, CIN, and cervical cancer tissue. All the results agreed that the expression of miR-145 in CIN samples was downregulated when compared to normal tissue, and the expression was further downregulated when reaching the cancer stage except in Wilting’s study. In Wilting’s study [107], the decrease of miR-145 was classified as ‘early transient’ altered miRNA expression, which means that miR-145 was significantly differentially expressed in CIN2–3 compared with normal tissue, but the difference decreased when reaching SCCs. This observation differs from those in the other three studies. Nevertheless, the decrease of miR-145 in CIN when compared to normal tissue was obvious. The early involvement of miR-145 in cervical cell transformation was proven by Martinez’s study, which employed multiple cell lines derived from low-grade CIN1 lesions [37]. Two of the cell lines (20,861 and 201,402) contained integrated HPV 16 DNA, while 20,863 only contained episomal HPV 16 DNA. The expression of miR-145 was found to be consistently downregulated in all three cell lines when compared to the normal cervix, but to different extents, with 20,863 showing the lowest level of miR-145. This result implies that the downregulation of miR-145 occurred even before the integration of the HPV viral genome into the host genome. E1 could initiate the viral DNA replication from episomal HPV [108]. Taking all evidence together, we propose that miR-145 may act on E1 to control the early replication of HPV when HPV enters the cells. When the HPV genome has been integrated fully into the host genome, the expression of miR-145 further decreased [37], which may facilitate the E1 function in both integrated and episomal replication. This phenomenon was not limited to HPV 16-infected cells but was also found in HPV-18-infected cells. The 8-mer seed sequences for miR-145 could be found in the coding regions of E1 in most HPV types, not just limited to HPV-31, suggesting its importance in viral function. KLF-4 is another target of miR-145. It is a transcription factor which contributes to stem cell pluripotency [109]. The interaction of miR-145 with KLF-4 contributes to the control of the viral life cycle during differentiation. 

#### 4.1.3. miR-34a

The expression of miR-34a precursor pri-miR-34a was first found to be downregulated in CIN in 2010 [59]. Infection with HPVs, especially HPV 16, contributes to the changes in miR-34a expression [60,110]. The dysregulation of miR-34a belongs to one of the very early changes in the development of cervical pre-cancer. In normal cervix tissue, infection with HPV leads to a downregulation of pri-miR-34a. The changes in the expression of miR-34a happened even before the morphological changes to the cervical cells [59]. CIN tissue with HPV infection also showed a lower expression of pri-miR-34a when compared to those without. The E6-p53-miR-34a pathway was validated in SiHa, 293T [59], Caski, HeLa, and C33A [24] cells but not in human samples. Nevertheless, the participation of the pathway in HeLa cells suggested that HPV 18 also controls the E6—p53—miR-34a pathway. In human samples, the expression of miR-34a showed a continuous downregulation from normal to LG-CIN to HG-CIN in exfoliated cells [14] and FFPE tissue [60]. Contradicting results were observed in Wilting [107] and Ribeiro’s [110] studies. In Wilting’s study, the expression of miR-34a increased from normal to CIN, while in Ribeiro’s study, the expression did not show significant changes when CIN conditions were compared with normal tissue. One of the explanations for this discrepancy is the lack of consideration for HPV status when analyzing the results. Low-risk HPV (lrHPV) does not degrade p53, and the expression of miR-34a is not affected if the human samples are infected by lrHPV [111]. In Ribeiro’s study, CIN samples containing hrHPV and lrHPV were pooled together for analysis, although > 75% of the CIN samples were hrHPV positive. However, the above explanation could not explain the observation in Wilting’s study. In Wilting’s study, all samples except one contained hrHPV. Lacking consideration of HPV infection status may not be the major factor contributing to the discrepancy. We believe p53 is not the sole regulator of miR-34a. MiR-34a may participate in other cellular processes related to HPV etiology. In Section 3.4.2, overexpression of miR-34a induced γH2A.X by suppressing PACS-1 in HeLa cells. Upon infection with HPVs, DDR is triggered, which might alter the expression of miR-34a for the induction of γH2A.X expression. The balance between the DDR and E6-p53 pathways determines the level of miR-34a present in the samples. Therefore, the expression of miR-34 highly depends on the stage of the transformation.

#### 4.1.4. miR-9

MiR-9 was found to be upregulated in CIN tissue [17,35,49] and serum [21,22] when compared to the normal samples. MiR-9 binds to 3′ UTR of FOXO3, one of the effectors in DDR, to downregulate the expression of FOXO3 [112]. In breast cancer, the expression of FOXO3 was negatively correlated to the expression of proteins (RAD50, Ku89, NBS, DNA-PK, PARP, and Mre11) involved in the DDR [113]. FOXO3 inhibits DDR and induces p53-dependent apoptosis [113]. The increase of miR-9 in CIN tissue may be related to the increase of DDR in the early cervical cell transformation. However, until now, no direct proof has been observed. On top of its possible participation in the DDR, miR-9 could be related to the differentiation in the early transformation process. A group from Washington University studied the relationship between miR-9 and cervical cancer using a bioinformatic approach. They performed gene ontology (GO) enrichment analysis to investigate the role of miR-9 expression in cervical cancer development. The results of the GO enrichment analysis suggested that miR-9 might coordinate tumour cell metabolism [114]. In terms of chromosome state, it was reported that the overexpression of miR-9 in CIN was associated with chromosomal gain of chromosome 1q in CIN samples [96]. As almost all the CIN samples with the 1q gain were hrHPV positive, it is anticipated that in hrHPV-mediated carcinogenesis, miR-9 serves as an oncogene. In the non-tumorigenic HPV 16-immortalized keratinocytes cell line, FK16, there was an increase in miR-9 expression from early to late passages of this cell line, implying that the upregulation of miR-9 is highly related to HPV 16. The increase in miR-9 expression led to an increase in cell viability, anchorage-dependent growth, and migration. Moreover, in organotypic raft cultures, miR-9 inhibited cell differentiation and increased cell proliferation during the early stage of cervical cell transformation. MiR-9 caused a decrease in cytokeratin-10 expression and a reduction in epithelial differentiation and enhanced proliferation of FK16 resulted [115]. The inhibition of the differentiation process posed by HPV E6 and E7 protein is essential for HPV replication and progression [115]. Indeed, HPV DNA-positive keratinocyte cells shared a comparable abnormal morphology with CIN. Therefore, the above experiment with keratinocyte cells implies that miR-9 could inhibit the differentiation during the CIN stage and facilitate proliferation of the transformed cells. However, certain knowledge of whether miR-9 interacted with E6 and E7 protein to cause such changes was elusive. 

#### 4.1.5. miR-21

Many studies on miR-21 focused on its changes of expression in invasive cervical cancer. Several studies agreed that there was a progressive increase of miR-21 from normal to CIN to cervical cancer [96,116,117]. Rationally, upon infection with HPV, the level of miR-21 would increase. However, the same trend was not observed in the HPV+ve/HPV-ve normal samples. The expression of miR-21 in normal cervical tissue infected with HPV was lower than those without HPV infection [118]. In the same study, the expression of miR-21 in CIN samples did not show a difference when compared to normal tissue. In Zeng et al.’s study [35], the expression of miR-21 in LSIL was lower than in normal tissue. The discrepancy between studies and the downregulation of miR-21 in HPV+ normal tissue implies that miR-21 functions differently in the initial stage of cervical cell transformation. Tumour-suppressor PDCD4 is a target protein regulated by miR-21. The 3′UTR of PDCD4 could be targeted directly by miR-21 as determined by luciferase reporter assay [119]. Multiple studies focused on the roles of PDCD4 in regulating programmed cell death, survival, and apoptosis. The increased level of miR-21 and the corresponding decreased level of PDCD4 promotes cell proliferation and suppresses cell death in the cervical cancer cells. However, during the initiation of cervical cell transformation by HPV, a decrease but not increase of miR-21 was observed. The decrease of miR-21 may be related to the HPV-induced DDR. PDCD4 has an important role in DDR [120,121]. The reduction of PDCD4 expression has been shown to impair the cellular DDR [120]. With the absence of p53, downregulation of PDCD4 hampered the DNA repairing process and decreased survival after UV treatment [121]. In the early stage of HPV infection, p53 started to degrade due to E6 protein expression. Together with the downregulation of miR-21 and an up-regulation of PDCD4, enhances cell survival through the DDR. HPV utilises the protein in DDR for self-replication. When reaching the cancerous stage, increased expression of miR-21 causes a decrease in PDCD4 and PTEN [122], resulting in higher proliferation and increased cell apoptosis. MiR-21 participated in the control of cell survival, proliferation, apoptosis, and invasion through the miR-21—PTEN—STAT3—MMPs [116], miR-21—PDCD4—mTORC-2/AKT [123], and miR-21-5p—TIMP3—VEGFA [124] pathways. However, whether these pathways are activated in the CIN stage is still not elucidated. 

**Figure 1:**
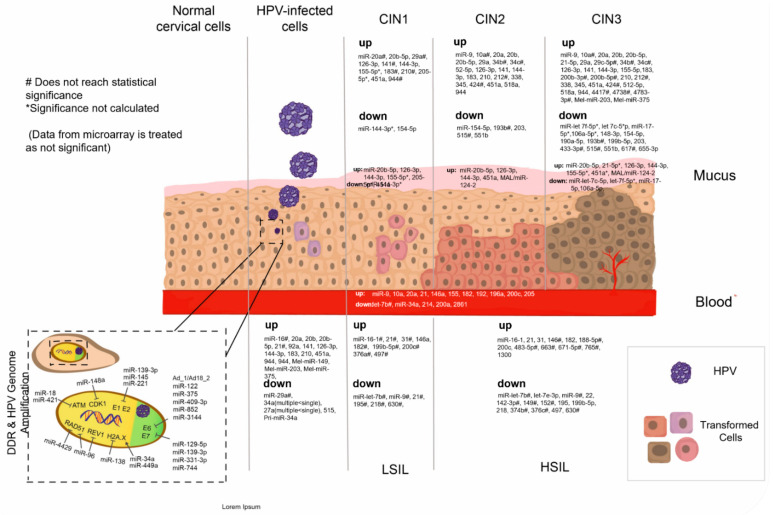
Summary of the dysregulated miRNAs in cervical cells infected with HPV, or at different cervical pre-cancerous stages, and miRNAs that mediated the DDR and HPV’s genome amplification in cervical cells. The up- and downregulations of miRNAs in CIN1-3, LSIL, and HSIL samples were determined by comparison with normal tissue. The dysregulated miRNAs found in blood samples from cervical pre-cancer patients were not segregated and were fitted into different cervical pre-cancer stages as those miRNAs were generally up- or downregulated in CIN or SIL (refer to Table 2). Data from microarray is treated as not significant; please refer to Table 2 for a list of the dysregulated miRNAs identified from microarray data. The schematic diagram was not drawn to scale for displaying the details of the components. For example, the purple spheres representing the HPV viral particles should be 1000 times smaller than the epithelial cells. It was enlarged to show the non-enveloped, icosahedral structure. The transformed squamous cells are colored in purple, red, light brown, and brown.

The dysregulated miRNAs found in cervical pre-cancer that are mentioned in our review are summarized in Figure 1. The miRNAs’ network for regulating DDR and genome amplification response in cervical cells is also illustrated in Figure 1.

## 5. Conclusions

Cervical pre-cancer consists of a spectrum of conditions. It is obvious that dysregulation of miRNAs was engaged in the early transformation of cervical pre-cancer. Indeed, miRNAs were involved in the initiation of multiple types of cancer, for example, ovarian cancer [125,126], lung cancer [127], and breast cancer [128]. Compared to later-stage cervical cancer, very few large-scale studies with the goal of uncovering miRNAs’ dysregulation during the early cervical transformation were observed. The previous studies have several short comings:

1. Selection of miRNA candidates was conducted by comparing cervical cancerous, but not specifically cervical pre-cancerous, tissue with normal tissue.
2. HPV status was always omitted during analysis.
3. Tissue was not micro-dissected in most studies.
4. There was a lack of specific analysis on microarray data and qRT-PCR data for cervical pre-cancer.
5. The studies did not consider the dysregulation of miRNAs together with the mechanisms involved in the early transformation of cervical pre-cancer.

The significance of this review includes:

1. Relates the dysregulation of miRNAs in cervical pre-cancer to the mechanisms posed by HPV infection in cervical cells. Understanding miRNAs’ involvement in HPV-related altered cell responses would help identify mechanism-based miRNA biomarkers. Mechanism-based biomarkers have a higher potential to identify the origin of diseases, reflect and segregate diseases types, and assess the stage of the disease [129].
2. Serves as a complete overview on the dysregulation of miRNAs in pre-cervical cancer when our review, He’s review, and Pardini’s review are taken together.
3. Compares the new studies with the old studies and locates overlapping miRNAs for further discussion.

Using a combination of miRNA biomarkers for the detection of cervical pre-cancer cells improves diagnostic sensitivity and specificity. Single miRNA biomarkers for detecting cervical pre-cancer are not satisfactory. The strategy of applying a combination of miRNA biomarkers could attain high differentiation power in segregating cervical pre-cancer from normal tissue or even different stages of cervical pre-cancer. Next, many studies focus on the diagnosis of cervical cancer but not cervical pre-cancer. Indeed, cervical pre-cancer progresses slowly. It usually takes several to ten years to progress from CIN1 to cervical cancer. The increase in the coverage of cervical pre-cancer screening efficiently reduces the need of the detection and treatment of cervical cancer. We hope that in the future, there are more research studies focusing on the detection of cervical pre-cancer. In addition, we observed that very different profiles of dysregulation of miRNAs were found in SIL and CIN tissue, although LSIL and HSIL are commonly equal to CIN1 and CIN2/3, respectively. The reasons for the discrepancy are still elusive. The difference in the methods used for the collection of cells could be one of the explanations. 

In future research, the level of miR-375, 145, 34-a, 9, and 21 in tissue, for example, blood or vaginal mucus, from cervical pre-cancer patients shall be investigated. The diagnostic power of this panel of miRNAs shall be determined. Second, the factors contributing to the discrepancy in the results observed for SIL and CIN samples shall be studied. Third, the relationship between the dysregulation of miRNAs and the DDR response shall be studied in the context of cervical pre-cancer initiation, but not only from the view of sensitization or resistance to drug or radiation therapy. Forth, extracting the microarray results in the previous studies, which include cervical pre-cancer samples for re-analysis, is encouraged. The re-analysis could help select miRNAs which are closely related to the initial cervical transformation. 

## Figures and Tables

**Table 1:** The dysregulated miRNAs appearing in the more recent studies were compared with those appearing in He and Pardini’s systematic reviews.

MiRNAs	Systematic Review (He Y et al.)	Systematic Review (Pardini B et al.)	More Recent Studies	
**Dysregulation in SIL**				
miR-21	•	•	•	Okoye JO et al., 2019 [13]
miR-146a	•	•	•	
miR-182		•	•	
miR-200c	•		•	
miR-let-7b		•	•	
**Dysregulation in CIN**				
miR-375	•	•	•	Tian Q et al., 2014 [14]
miR-21	•	•	•	Kawai et al., 2018 [15]
miR-126-3p		•	•	
miR-155-5p	•	•	•	
miR-205-5p		•	•	
miR-20a		•	•	Liu SS et al., 2018 [16]
miR-92a	•	•	•	
miR-210	•	•	•	
miR-199b-5p		•	•	Han MS et al., 2018 [17]
miR-29c	•	•^#^	•	Zhao W et al., 2020 [18]
miR-617	•	•	•	
miR-20b	•	•	•	Szekerczés T et al., 2020 [19]
miR-212		•	•	
**Biomarker for cervical pre-cancer**				
miR-497	•	•	•	Zhang Y et al., 2015 [20]
miR-10a	•	•	•	Xin F et al., 2016 [21]
miR-196a	•	•	•	
miR-146a	•	•	•	Okoye JO et al., 2019 [13]
miR-155	•	•	•	
miR-182		•	•	
miR-200c	•		•	
miR-let-7b		•	•	
miR-9	•	•	•	Farzanehpour M et al., 2019 [22]
miR-192	•	•	•	
miR-205		•	•	
miR-21	•	•	•	Wang H et al., 2019 [23]
miR-34a	•^#^	•^#^	•	
miR-200a	•	•	•	
**HPV infection**				
miR-34a	•^#^	•^#^	•	Wang X et al., 2009 [24]
miR-375	•	•	•	Lu H et al., 2016 [25] Morel A et al., 2017 [26]
miR-20a		•	•	Liu SS et al., 2018 [16]
miR-141	•	•	•	
miR-210	•	•	•	
miR-20b-5p	•	•	•	Kawai S. et al., 2018 [15]
miR-21	•	•	•	Zamani S et al., 2019 [27]
miR-29a	•^#^	•^#^	•	
miR-221		•^#^	•	Lu H & Gu X, 2019 [28]
miR-20b	•	•	•	Szekerczes T et al., 2020 [19]
miR-16	•	•	•	Zubillaga-Guerrero, M.I. et al., 2020 [6]
**DNA Damage Response**				
miR-148a	•^#^	•^#^	Wang C et al., 2020 [29]	
miR-34a	•^#^	•^#^	Veena M.S. et al., 2020 [30]	

• miRNA upregulation during cervical pre-cancer progression or upon HPV infection. • miRNA downregulation during cervical pre-cancer progression or upon HPV infection. •^#^ Conflicting miRNA expression level changes among different studies analyzed by He Y et al. and Pardini B et al. systematic reviews.

**Table 2:** MiRNAs dysregulated in the cervical pre-cancer stage in different human sample types in Section 2.

Reference	Sample Type	Sample Number	MiRNAs	Change of Expression Level	Study Method
**Dysregulation in SIL**					
Zeng et al., 2015 [35]	FFPE cervical tissue	Screening: Normal (*n* = 3), LSIL (*n* = 3), HSIL (*n* = 3), SCC (*n* = 3) Validation: Normal (*n* = 13), LSIL (*n* = 15), HSIL (*n* = 35), SCC (*n* = 40)	miR-31 #, miR-199b-5p #, miR-376a #, miR-497 #,	Up (LSIL)	Microarray (top 6 dysregulated), qRT-PCR
			miR-31, miR-188-5p, miR-483-5p, miR-663, miR-671-5p, miR-765, miR-1300	Up (HSIL)	
			miR-9 #, miR-21 #, miR-195 #, miR-218 #, miR-630 #	Down (LSIL)	
			miR-9 #, miR-142-3p, miR-149, miR-152, miR-195, miR-199b-5p, miR-218, miR-374b, miR-376c, miR-497, miR-630 #	Down (HSIL)	
Chen X et al., 2016 [31]	Frozen cerival tissue	Normal (*n* = 26), HSIL (*n* = 37), cervical carcinoma (*n* = 101)	miR-let-7e-3p	Down (HSIL, cervical carcinoma)	qRT-PCR
Wongjampa W et al., 2018 [33]	Fresh, FFPE cervical tissue	Tissue: LSIL (*n* = 22), HSIL (*n* = 20), SCC (*n* = 30). HPV −ve NSIL (*n* = 30) FFPE: HR-HPV+ve HSIL (*n* = 14), SCC (*n* = 11)	miR-22	Down (HSIL, SCC)	Microdissection, qRT-PCR
Okoye et al., 2019 [13]	Liquid-based cytology and preserved cervical cells	Normal (*n* = 159), cervicitis (*n* = 46), ASCUS (*n* = 46), LSIL (*n* = 40), HSIL (*n* = 28), SCC (*n* = 10)	miR-21, miR-146 #, miR-182, miR-200c,	Up (HSIL)	qRT-PCR
			miR-21 #, miR-146a, miR-182 #, miR-200c #	Up (LSIL)	
			miR-let-7b	Down (HSIL #, LSIL #)	
Zubillaga-Guerrero MI et al., 2020 [6]	Liquid-based cervical tissue	HPV+/−ve NSIL (*n* = 20), LSIL (*n* = 20), HSIL (*n* = 20), SCC with HR- HPV (*n* = 20)	miR-16-1	Up (LSIL #, HSIL)	qRT-PCR
**Dysregulation in CIN**					
Cheung TH et al., 2012 [49]	Cervical biopsy specimen	Normal (*n* = 9), HG-CIN (*n* = 48), SCC (*n* = 51)	miR-9, miR-10a #, miR-20b, miR-34b #, miR-34c #, miR-338, miR-345, miR-424 #, miR-512-5p, miR-518a	Up (HG-CIN > Normal)	Microdissection, qRT-PCR
		Independent cohort: CIN2, (*n* = 6), CIN3 (*n* = 18)	miR-193b#, miR-203	Down (HG-CIN < Normal)	
Wilting SM et al., 2013 [50]	FFPE cervical tissue	Normal (*n* = 16), CIN1 (*n* = 4), CIN3 (*n* = 13), SCC (*n* = 20)	miR-203, miR-375	Methylation levels: Up (CIN3, SCC)	Quantitative methylation specific PCR
Liu SS et al., 2018 [16]	Paraffin-embedded tissue, Frozen tissue	Screening: Normal (*n* = 5), LG-CIN (*n* = 5), HG-CIN (*n* = 5), CC (*n* = 5) Validation: Normal (*n* = 145), LG-CIN (*n* = 239), HG-CIN (*n* = 285), CC (*n* = 58)	miR-20a, miR-92a, miR-141, miR-183, miR-210, miR-944	Up (LG-CIN#), (HG-CIN)	Microarray, qRT-PCR
			miR-92a, miR-183	Up (from LG-CIN to HG-CIN) #	
			miR-20a, miR-141, miR-210, miR-944	Down (from LG-CIN to HG-CIN) #	
Kawai S et al., 2018 [15]	Frozen cervical mucus	Normal (*n* = 16), CIN1 (*n* = 11), CIN3 (*n* = 29), SCC (*n* = 19), and AD (*n* = 11)	miR-20b-5p, miR-126-3p, miR-144-3p miR-451a	Up (CIN1, 2, 3, SCC)	Microarray, qRT-PCR
			miR-155-5p *, miR-205-5p *	Up (CIN1 > Normal)	
			miR-144-3p *	Down (CIN1 < Normal)	
			miR-21-5p *, miR-144-3p *, miR-155-5p *, miR-451a *	Up (CIN3 > Normal)	
			miR-17-5p *, miR-106a-5p *, miR-let-7c-5p *, miR-let-7f-5p *	Down (CIN3 < Normal)	
Lukic A et al., 2018 [51]	FFPE cervical tissue	Normal (*n* = 10), condylomas (*n* = 18), CIN1 (*n* = 8), CIN2,3 (*n* = 14)	miR-551b	Down (CIN2, 3)	qRT-PCR
Han MS et al., 2018 [17]	FFPE cervical tissue Frozen cervical tissue	HPV 16 +ve normal cervix (*n* = 3) HPV 16 +ve cervical carcinoma (*n* = 3)	miR-148-3p, miR-190a-5p, miR-199b-5p, miR-655-3p	Down (CIN3, cancer IA group) > (HPV−ve− normal, HPV+ normal, HPV−ve cancer)	Hybridization, qPCR
Zhao W et al., 2020 [18]	Frozen cervical tissue	HPV 16 +ve normal (*n* = 35), CIN1 (*n* = 31), CIN2/3 (*n* = 33), SCC (*n* = 31)	miR-29c-5p, miR-200b-3p, miR-200b-5p, miR-4417, miR-4738, miR-4783-3p	Up (CIN3 > Normal)	Microarray, qRT-PCR
			miR-433-3p, miR-617	Down (CIN3 < Normal)	
			miR-154-5p	Down (CIN1, 2/3, CC)	
Szekerczés T et al., 2020 [19]	Paired FFPE cervical tissue	Screnning: 10 paired (normal/diseased) CIN1, 2, 3, CIS Validation: 22 paird CIN2–3 and surrounding normal tissue	miR-20b, miR-212 #	Up (CIN2,3) #	Microarray, Microdissection, qRT-PCR
			miR-515 #	Down (CIN2, 3) #	
**Biomarker for cervical pre-cancer**					
Verhoef VM et al., 2014 [56]	Lavage	High-risk HPV(*n* = 772), ≤ CIN1 (*n* = 547), CIN2 (*n* = 78), CIN3 (*n* = 134), SCC (*n* = 13)	MAL/miR-124-2 methylation	Methylation levels: Up (CIN2, 3)	Quantitative methylation specific PCR
Liu P et al., 2015 [55]	Frozen Serum	Normal (*n* = 50), CIN (*n* = 86), CC (*n* = 105)	miR-196a	Up (CIN, CC)	qRT-PCR
Zhang Y et al., 2015 [20]	Frozen Serum	Normal (*n* = 213), CIN1 (*n* = 27), CIN2 (*n* = 120), CIN3 (*n* = 39), CC (*n* = 184)	miR-16-2, miR-497	Up (CC)	qRT-PCR
			miR-195	Down (CC)	
			miR-2861	Down (CIN, CC)	
Xin F et al., 2016 [21]	Frozen Serum	Normal (*n* = 60), CIN (*n* = 126)	miR-9, miR-10a, miR-20a, miR-196a	Up (CIN)	qRT-PCR
Kawai S et al., 2018 [15]	Frozen cervical mucus	Normal (*n* = 56), CIN1 (*n* = 19), CIN2 (*n* = 33), CIN3 (*n* = 43), SCC (*n* = 35), and AD (*n* = 19); NILM (*n* = 39), LSIL (*n* = 21), HSIL (*n* = 75), SCC (*n* = 24), AD (*n* = 16)	miR-20b-5p, miR-126-3p, miR-144-3p, miR-451a	Up (CIN1, 2, 3, SCC)	qRT-PCR
			miR-155-5p *, miR-205-5p *	Up (CIN1 > Normal)	
			miR-144-3p *	Down (CIN1 < Normal)	
			miR-21-5p *, miR-144-3p *, miR-155-5p *, miR-451a *	Up (CIN3 > Normal)	
			miR-17-5p *, miR-106a-5p *, miR-let-7c-5p *, miR-let-7f-5p *	Down (CIN3 < Normal)	
Farzanehpour M et al., 2019 [22]	Frozen Serum	CIN (*n* = 186)	miR-9, miR-192, miR-205	Up (CIN)	qRT-PCR
Okoye et al., 2019 [13]	Frozen Serum	Normal (*n* = 159), cervicitis (*n* = 46), ASCUS (*n* = 46), LSIL (*n* = 40), HSIL (*n* = 28), SCC (*n* = 10)	miR-21, miR-146a, miR-155, miR-182, miR-200c	Up (SIL)	qRT-PCR
			miR-let-7b	Down (SIL #)	
Wang H et al., 2019 [23]	Frozen Plasma	Normal (*n* = 42), HPV+ve (*n* = 31), CIN1 (*n* = 19), CIN2 (*n* = 54), CIN3 (*n* = 71), CC (*n* = 15)	miR-21	Up (CIN1 #, 2, 3)	qRT-PCR
			miR-34a, miR-200a, miR-214	Down (CIN1 #, 2, 3)	

# Does not reach statistical significance. NILM = negative for intraepithelial lesions and malignancy. CIS = carcinoma in situ. NSIL = no sign of SIL. BROWN—from qPCR data. PINK—from microarray data, treated as not significance. * = Significance not calculated.

**Table 3:** MiRNAs dysregulated in the cervical pre-cancer stage owing to HPV infections.

Reference	Sample Type	Sample Number	MiRNAs	Change of Expression Level in Cervical Pre-Cancerous Tissue	Study Method
**HPV**					
Li B et al., 2010 [59]	Cervical tissues	Normal without HPV(*n* = 32), Normal with HPV (*n* = 32), CIN with HPV (*n* = 32), CIN without HPV (*n* = 12), CC (*n* = 32)	Pri-miR-34a	Down (normal and CIN with hrHPV)	Semi-qRT-PCR
Wilting SM et al., 2013 [50]	HPV-immortalized keratinocytes		miR-149	Up methylation (HG-CIN HPV-immortalized)	qRT-PCR
	FFPE cervical tissue	Normal (*n* = 16), CIN1 (*n* = 4—50% hrHPV-positive), CIN3 (*n* = 13—92% hrHPV-positive), SCC HPV-positive (*n* = 20)	miR-203, miR-375	Up methylation (CIN3 and SCC mostly HPV-positive)	
	Cevical crapes	Normal with HPV (*n* = 13), HG-CIN with HPV (*n* = 17)	miR-203	Up methylation (HG-CIN and abnormal with hrHPV-positive)	
Gocze K et al., 2015 [60]	FFPE tissue	CIN1 (*n* = 30), CIN2 (*n* = 10), CIN3 (*n* = 20), SCC (*n* = 38), HPV-positive in each category (*n* = 98)	miR-34a, pri-miR-34	Down (CIN HPV 16-positive)	qRT-PCR
			miR-27a, miR-34a	Down (CIN2-CIN3 with multiple HPV) compared to with single HPV infection	
Liu SS et al., 2018 [16]	Liquid nitrogen stored cervical tissues	Normal (*n* = 145—24% hrHPV-positive), HG-CIN (*n* = 285—82% hrHPV-positive), LG-CIN (*n* = 239—64% hrHPV-positive)	miR-20a, miR-141, miR-210, miR-944	Up (normal with HPV)	Microarray, qPCR
			miR-92a, miR-183, miR-944	Up (HG-CIN with HPV 16)	
Kawai S et al., 2018 [15]	Frozen cervical mucus	Normal (*n* = 56), CIN1 (*n* = 19), CIN2 (*n* = 33), CIN3 (*n* = 43), SCC (*n* = 35), and AD (*n* = 19); NILM (*n* = 39), LSIL (*n* = 21), HSIL (*n* = 75), SCC (*n* = 24), AD (*n* = 16)	miR-20b-5p, miR-126-3p, miR-144-3p, miR-451a	Up (HPV 16/18 infection vs. other HPV infection status)	qRT-PCR
Zamani S et al., 2019 [27]	Liquid-based Cytology Samples (LBCs)	Normal without HPV (*n* = 46), Normal with HPV (*n* = 43), CIN1 (*n* = 9), CIN2 (*n* = 6)	miR-21 #	Up (HPV-positive)	qRT-PCR
			miR-29a #	Down (HPV-positive)	
Szekerczes T et al., 2020 [19]	FFPE tissue	Normal (n=54), CIN1(*n* = 10), CIN2 (*n* = 54), CIN3 (*n* = 54), CIS (*n* = 10), HSIL/CIN2–3 with HPV (*n* = 13)	miR-20b	Up (HSIL/CIN2–3 HPV-positive)	qRT-PCR
			miR-515	Down (HSIL/CIN2–3 HPV-positive)	
Zubillaga- Guerrero MI et al., 2020 [6]	Liquid-based cytology cervical sample	Normal without HPV (*n* = 20), LSIL (*n* = 20), HSIL (*n* = 20), SCC HrHPV (*n* = 20)	miR-16	Up (SIL and SCC with mixed HPV state) (HPV positive)	qRT-PCR

# Does not reach statistical significance.

**Table 4:** Regulations on HPV encoded protein by miRNAs.

Regulation in HPV	Corresponding miR	Protein Target	References
**Inhibit E1–E2**	miR-139-3p	n.a.	Sannigrahi MK et al., 2017 [62]
	miR-145	KLF-4	Gunasekharan V & Laimins LA, 2013 [63]
	miR-221	IFN(SOSC)	Lu HK & Xin Gu, 2019 [28]
**Inhibit E6**	Ad16_1/Ad18_2	n.a.	Bonetta AC et al., 2015 [64]
	miR-122		He J et al., 2014 [65]
	miR-375		Morel A et al., 2017 [26]
	miR-409-3p		Sommerova L et al., 2019 [66]
**Promote E6 splicing**	miR-875	EGFR	Li Y et al., 2018 [67]
	miR-3144		
**Inhibit E6–E7**	miR-129-5p	SP1	Zhang J et al., 2013 [68]
	hsa-miR-139-3p	n.a.	Sannigrahi MK et al., 2017 [62]
	miR-331-3p	NRP2	Fujii T et al., 2016 [69]
	miR-744	ARHGAP5	Li et al., 2019 [70]

**Table 5:** MiRNAs associated with the DDR of cervical cells.

Phase of DDR	DDR Proteins	Corresponding miR	References
**Initiation**	ATM	miR-18 miR-421	Liu S et al., 2015 [84] Hu H et al., 2010 [83]
	CDK1	miR-148a	Wang C et al., 2020 [29]
**DSB marker**	H2A.X	miR-138	Wang Y et al., 2011 [87]
	γH2A.X	miR-34a miR-449a	Veena MS et al., 2020 [30]
**Repairing**	RAD51	miR-96 miR-4429	Sun H et al., 2020 [93] Wang Y et al., 2012 [92]

## Data Availability

Not applicable.
